# Trust, Media Credibility, Social Ties, and the Intention to Share towards Information Verification in an Age of Fake News

**DOI:** 10.3390/bs12020051

**Published:** 2022-02-16

**Authors:** Przemysław Majerczak, Artur Strzelecki

**Affiliations:** Department of Informatics, University of Economics in Katowice, 40-287 Katowice, Poland; przemyslaw.majerczak@edu.uekat.pl

**Keywords:** fake news, social media, internet

## Abstract

Social media is now the primary form of communication between internet users and has soared in popularity, which has directly impacted the spread of the phenomenon of fake news. Fake news is not only a widespread phenomenon; it is also problematic and dangerous for society. The aim of this study is to understand the phenomenon of fake news better. The study utilised a structural modelling equation in order to identify how Polish society perceives the problem of fake news and assess the extent to which it trusts content that is published on the internet. The key goal was to determine what factors have the most significant influence on the verification of information being viewed on the internet. By deploying the partial least squares method of validation, SmartPLS3 software was used to process the survey results. The strongest positive effect on information verification behaviour was found to be fake news awareness, which was followed by the intention to share information. The research did not consider any clear connections that may exist between the nature of fake news and its recipient; however, much of the fake news that appears on the internet is political in nature. The study can be used by news reporting companies and provides preliminary information for developers responsible for running social media sites as well as users who want to combat and limit the spread of fake news online. This study expands on the available literature related to fake news by identifying the effects on information verification behaviour of fake news awareness and the intention to share data.

## 1. Introduction

Fake news is a neologism that makes it difficult to place in a definitional framework [1]; as a result, various definitions for fake news have appeared in publications. Although these differ depending on interpretation and perspective, they all have one element in common: the foundation that binds most definitions together is the falsity of the information and the desire to imitate it superficially to give the impression of real news. Many authors also draw attention to the fact that the creation and dissemination of fake news often bring ideological or material benefits to its author [2]. A desire to mislead the viewer in order to provoke a specific emotion or take a particular stance on a given issue is also highlighted. Since this phenomenon is not based on facts but on arousing emotions, it is often encountered when stereotyping or in political, religious, or other highly controversial issues [3]. Fake news mainly aims to shock and incite social discussion and conflict. According to A. Gelfert, fake news is a genre of disinformation, i.e., false information that is intended to mislead its reader or create a particular view on a given topic [1].

There are several types of disinformation, which are divided according to content [4]. The first is fabricated content where the information provided is completely new, and the content is false by design. This can be either textual or visual and is supported by, for example, graphics. Another popular form of disinformation is manipulated content. This may be authentic content that is changed to deceive the reader, or imposter content, where the source is legitimate, but the content has been manipulated to misinform. Another type of misinformation is the inverse of false context. In this case, real information is given in a false context. There is also misleading content. Here, the user is misled by appropriately cropped images or selectively chosen quotations that create a completely different context. There are also false connections, where headlines have a link that can be clicked on, but after opening the given link, the headlines have nothing to do with the content shown and are in no way supported by the text that is visible on the page. The last type of misinformation is satire and parody. In social media, these are pages whose main premise is to parody; the content that appears on these pages may be perceived as satirical and can often be misunderstood [4].

When analysing fake news, it is important to question the origin of this phenomenon and consider why false information spreads so quickly. In this research, we address the question of why fake news is more popular and more often read than real content [5]. Vosoughi et al. (2018) addressed the spread of true and false news online and examined the differences in the spread of both true and false news on Twitter between 2006 and 2017 [6]. Data that covered 126,000 posts that were published on this social network by nearly 3 million people over 4.5 million times showed that fake news spread at a much faster rate than real content. They produced a model estimation of the likelihood of content being retweeted. The results showed that false information was up to 70% more likely to be retransmitted than true information [6]. The study also confirmed how quickly falsehoods spread: the time it takes for fake news to reach 1500 people is about six times shorter than that of real news.

People have always been fascinated by what is new and previously unheard of, which is what fake news is [7]. In the age of social media, there is a direct correlation between being the first to report news and being perceived as better informed. Fake news also reaches much deeper into the public’s subconscious; it arouses greater emotion and more involvement, and, consequently, it is shared instantly. This was confirmed during the 2016 U.S. presidential election campaign when the internet was flooded with information claiming that Pope Francis had endorsed presidential candidate Donald Trump. To make this information real, the fake news authors also inserted an altered photograph that showed the Pope and Donald Trump together [8]. More than 10 million people saw this post on Facebook, which shows what a large reach social media has and, therefore, how dangerous it is.

This example is one of many fake news stories that appeared during the 2016 U.S. presidential election campaign. After the election, numerous studies were conducted that demonstrated, among other things, the exposure of different social groups to fake news [8,9]. Accordingly, those who were perceived as being characteristically more susceptible to fake news during this period were identified. Typically, they were older, conservative and highly involved in national politics [10]. It is worrying that fake content that is shared on social media has a much larger audience than real news published in the press.

Authors of fake news usually have a specific goal in publishing it. According to Gelfert [1], “fake news is false or misleading claims as news, where these are misleading by design”. There may be several reasons for the appearance of fake news on the internet. The first is financial in nature. A fake news authors’ primary goal is that the user clicks on the link of the fake news being shared in order to create website traffic. Then, the reader is redirected to a website full of advertisements, thanks to which the author of the fake news site gains income. In this scenario, the function of a fake news site is that of clickbait, e.g., exciting news stories such as the apparent death of a famous person [11].

The second reason arises from ideological convictions and political expediency. The authors of fake news want to win as much support as possible from society. This occurs most often during election campaigns when every vote is important and political supporters cannot win honestly. Apart from false information, which seemingly puts its authors in a better light, fake news often appears simultaneously in order to harm other parties or particular politicians. Another reason for publishing false information may be for the purpose of propaganda [12], which serves to arouse public discussion and controversy.

The lack of research among people who would like to share their experiences with verification of fake news represents a literature gap. The referenced studies, if they were carried out among people, were conducted with the use of other study methods than are proposed in this study. In this work, we would like to fill this gap by providing unbiased data from internet users on how they verify news on the internet regarding the type of source. With this in mind, we prepared a study among internet users to fill the current gap in the literature. By using the well-established PLS-SEM modelling, this paper aims to describe internet user information verification and intentions to share. The internet has different expectations of social media credibility and trust in people online. The study explores which variables are the best predictors of users’ intention to verify information online and determines how users perceive fake news awareness.

This study’s contribution to general research on fake news awareness consists of collecting data from users who have used social media and have experience in online behaviour, and have encountered different types of risks associated with fake news. The state-of-the-art research contains a model of external variables that impact information verification. The data were gathered through a questionnaire survey for users of different online social media platforms. The research’s main finding is that the best predictor of users’ information verification is their fake news awareness, followed by their intention to share. Furthermore, trust in people online, and social media credibility have a negative impact on information verification.

## 2. Literature Review

As key components of this online phenomenon, the importance of online bots and fake accounts, both of which are common in social media, should first be explained, followed by clickbait, filter bubbles and internet trolls. 

### 2.1. Internet Bots/Fake Accounts

Fake accounts and online bots are both common in social media; some fake news articles on the internet are published by internet bots, but it is impossible to say how many. The word comes from “robot”, which is an automated device that performs predetermined actions. Internet bots work in a very similar way. They carry out specific tasks that have been assigned to them by a human. The difference is that all these activities are carried out on online platforms. Web bots are programs that run continuously, formulate decisions, act on those decisions without human intervention, and can adapt to the context in which they operate [13].

There are many different types of web bots that can have a negative effect on users, depending on the purpose for which they were created [13]. The most common type of web bots are chatbots, which are used to conduct a dialogue between a human and a computer that operates in natural language using text or speech. They are designed to interact with the user directly or through an interface.

In this age of social media, socialbots are also popular. These are accounts that are controlled by a computer algorithm to create content and interact automatically with humans. Socialbots interact with users in various ways through these platforms and may be created specifically to manipulate or spread false information on social media [14]. One example of a socialbot is a Twitter bot, which operates on the Twitter platform and can perform actions such as liking posts, following accounts, or sending private messages to other users. Twitter bots are also used to publish fake news. It is estimated that as much as 9% to 15% of active accounts on this social network are bots [2]. According to Gorwa (2017), as much as every third post published on Polish Twitter is shared using fake accounts [15]. By being fully automatic, online bots are mainly used for hate propagation, spamming, and compromising specific individuals or entire groups [15]. 

Social media bots play a significant role in sharing articles from sources as soon as they appear online. Their function is to amplify content before the article goes viral. Social media bots strongly support sources with a low level of credibility, and thus these sources become more persuasive. Furthermore, readers believe the information that is sent by bots and then share it, thus indicating that people are relatively easy to manipulate [16].

### 2.2. Clickbait

Clickbait refers to fake web content that attracts the attention of readers with the help of deceptive, shocking, or even impossible headlines. A clickbait post is designed to interest as many users as possible by using keywords or emotional tags [17]. It often uses ambiguity to persuade the user to click on a link. The main goal of those who use clickbait is to increase the number of users visiting their website and thus generate more advertising revenue [18]. Clickbait titles are a common phenomenon found on social media that serve to arouse curiosity among users. Clickbait can be annoying for readers, who feel let down by the promise of what they would discover by clicking on the headline compared to what they actually read in the content. Clickbait also causes the proliferation of fake news on the internet as it is shared by users who have not first read the content [19].

Clickbait detection methods are difficult to implement because information systems have difficulty distinguishing clickbait headlines from real headlines due to the limited availability of information. Existing clickbait detection algorithms can be divided into those based on lexical similarity and those that use machine learning [20,21]. Algorithms that operate on the basis of lexical similarity are designed to detect clickbait that is based on semantic similarities between headlines and their corresponding content. [19]. The Yahoo research team managed to develop one such algorithm using supervised machine learning. The research aimed to prove that there is a relationship between the informality of an article and clickbait. Therefore, several measures of textual formality were used to help identify clickbait [18,22].

### 2.3. Filter Bubbles

The filter or information bubble is based on an algorithm that targets specific users with selected content, which is chosen based on the user’s profile history [23]. The purpose is to make it easier for people to access the kind of information they are likely to want to see rather than content they would be less interested in [9]. 

In this scenario, the reader receives interesting content, which is a good thing. However, social media has been criticised for creating digital echo chambers in which users see content and posts that only agree with their existing beliefs [24]. This content, which may also be false, reinforces the beliefs and opinions an audience already holds while not allowing them to see that alternatives exist [23]. In 2015, Facebook conducted a study that showed that although this social network’s internal algorithm can select posts that confirm a user’s political beliefs, the effect of filter bubbles is mainly due to the user’s behaviour, such as how they click and search for specific content of interest. This shows that it is mainly the choices of the users themselves that creates this bubble rather than simply Facebook’s algorithms [24].

### 2.4. Internet Trolls

Internet trolls also manipulate users through social media. These are usually fake accounts whose aim is to irritate users and provoke arguments and conflicts between online communities. Internet trolls achieve this by posting content that is controversial, provocative, accusatory, or simply untrue. Often this is done just for fun in order to disrupt discussions and provoke emotional reactions [25]. Online trolling involves behaving in a deceptive or disruptive manner in a social media environment with no clearly defined purpose. If a person does not understand the troll’s intentions and falls into the troll’s trap, then the trolling can escalate even further [26].

### 2.5. Hypotheses Development

Social media is characterised by the involvement of the user in how their account is run. Consequently, individual members make decisions regarding the dissemination of published information. Research is being conducted to understand how decisions are made, including verification of sources’ veracity. Observations suggest that beliefs about the veracity of the information that is received through social media depend largely on the individuals who share it [27]. If a user has friends or observes people who come from completely different social backgrounds, then there is a high probability that they will encounter different points of view. The variety of opinions may cause the user to start questioning the information that emerges on social media. Therefore, when friends publish opposing information, the user should develop an awareness of fake news. Even if they are not fully able to determine what news is false, the very presence of contradictory views would indicate that at least some of the news is false. Through situations such as this, users have a better awareness of fake news. Based on the above, we propose the following hypothesis:

**Hypothesis** **1** **(H1).** The diversity of social ties positively affects fake news awareness.

Self-beliefs influence the online actions that users take. In social media, trust has emerged as an important governance mechanism that regulates the behaviour of network members [28,29]. If trust is present, a person may forego actions that they would normally perform in the absence of this relationship. It can be assumed that where there is a high level of trust, recipients of news refrain from verifying the source from which they obtain information because they rely on people who are considered trustworthy [30]. Based on the above, we propose the following hypothesis:

**Hypothesis** **2** **(H2).** Trust in people online negatively affects information verification.

How users perceive the authors of published news and whether these authors are an authority on a given subject is likely to influence whether users will verify the information they read. For the user, an authority is a person who is aware of fake news and has specific knowledge of situations in which a source of information is false. This authority, which other users trust, may be able to identify those media outlets that are unreliable sources of information [31]. Fake news awareness and social media credibility provide a measure of authority for the user. When individuals perceive that information reported by a particular source is misleading, they then perceive the source to be incompetent, at the very least. In a worst-case scenario, they may begin to question the positive intentions of that source. It is, therefore, most important for the authors of published news to be perceived as reliable sources of information. It can be argued that people with a higher level of awareness of fake news are more sceptical about the credibility of social media. Based on the above, we propose the following hypothesis:

**Hypothesis** **3** **(H3).** Fake news awareness has a negative impact on social media credibility.

Information verification is a response to the recipient’s awareness that the news may be misleading [32]. Studies show that users use different methods to determine the veracity of information. Consequently, some users are better able to detect fake news than others [33]. Correspondingly, social media members may have varying degrees of knowledge regarding fake news due to their skills and experience. When a news source reports the appearance of a piece of fake news and that it should not be believed, some people will simply forget about the item and fail to consider how it came about. However, more advanced users who are interested in the topic of fake news and want to detect inaccuracies will look for alternative sources of information in order to better understand this phenomenon [34]. These individuals are more aware of the occurrence of fake news and find it easier to identify; however, if a user is completely unaware of fake news, they are unlikely to take any measures to verify information viewed online. A user may seek information on fake news to satisfy a desire for additional knowledge. Simply by verifying information, such a person is aware of the possible existence of fake news. Therefore, people who are aware of fake news are more likely to attempt to verify the source than people who are less skilled at distinguishing true from false information. Based on the above, we propose the following hypothesis:

**Hypothesis** **4** **(H4).** Fake news awareness has a positive effect on information verification.

Research shows that users are increasingly assessing the reliability of the information on the internet. However, depending on the type of news, both the degree and the means of verification vary [33,35]. Undoubtedly, people who are sceptical about news appearing in different media outlets usually look for alternative sources of information [36]. Nowadays, more and more people are beginning to recognise the issue of the credibility of the information sources that they view in the media. This is due to various beliefs or biases towards news creators and the incompetence and manipulative ploys used by the media [8]. There is an increasing tendency to look for other sources to verify the information. Therefore, it is logical that the influx of fake news on social media increases scepticism about information sources and may encourage users to be more vigilant in verifying information. Based on the above, we propose the following hypothesis:

**Hypothesis** **5** **(H5).** Social media credibility has a negative impact on the degree of information verification.

There is a lot of research on social media sharing. This is directly related to the proliferation of news on social media [37]. Studies suggest that users tend to share information that they consider to be important or of a personal nature [38]. This behaviour can therefore be considered to have important implications for the degree of information verification. This situation is especially likely to occur when the news is in line with the ideological position taken by the user. In this case, the recipient is unlikely to verify the source or critically evaluate the information, as would probably have occurred in another situation. Thus, it can be observed that news received through social media significantly impacts subsequent decisions to disseminate such information. Online image is important; it is shaped not only by a user’s account profile but also by published posts. However, it is important to choose content carefully and truthfully because a user can easily be criticised or simply misinterpreted. It can be concluded that if a person reads a piece of news but does not intend to share it on their profile, there is much less chance that they will verify it as it does not directly affect their online image. However, spreading fake news may significantly damage a person’s credibility on the internet and considerably weaken their online image. This leads to the conclusion that people who have more of an intention to share information are more likely to verify the information they publish. Based on the above, we propose the following hypothesis:

**Hypothesis** **6** **(H6).** The intention to share information has a positive effect on the degree of information verification.

## 3. Method

These hypotheses show the direction of the variables’ interactions with each other. Next, each variable has items that are questions that are asked in the survey. The research technique that was employed involved using a questionnaire to collect responses. The questionnaire was shared on Polish Facebook community groups using Google Forms between 12 January 2021 and 31 January 2021. The survey consisted of 24 questions and a metric. All the questions used in the survey used a 7-point Likert scale. The questionnaire is in Appendix A.

**Figure 1:**
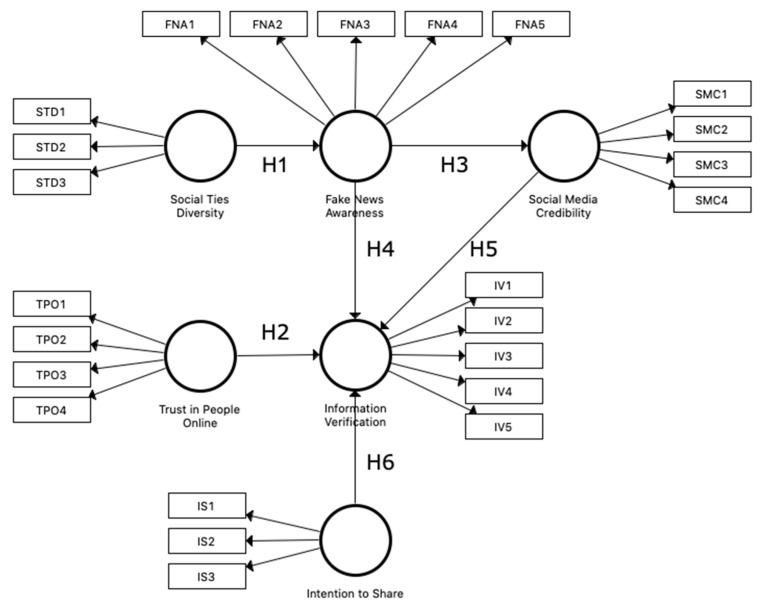
The basic model in SmartPLS.

Using a Polish group of respondents, the research that was undertaken was inspired by the work proposed by Torres et al. [39]. The various social media platforms rely heavily on the participatory engagement of their members because it is they who decide whether information should be disseminated. Therefore, it is important to gain a better understanding of how this decision occurs and the impact it has on related behaviours, such as information verification. This is crucial for understanding user interactions in social media environments [39]. The study investigates which factors have a stronger influence on users’ verification of information and what determines how we perceive fake news. We explore whether information verification is affected by social media credibility, fake news awareness, trust in people online, and intention to share. Figure 1 shows the basic model with variables and hypotheses.

This model was prepared using structural equation modelling (SEM). This is often used for statistical modelling in behavioural sciences. Structural equation modelling is a multivariate statistical analysis that involves determining the type and the strength of relationships. Two types of variables are used in SEM: endogenous and exogenous. Endogenous variables, also known as explanatory variables, are equivalent to dependent variables.

Exogenous (explanatory) variables are called independent variables; endogenous variables are calculated based on exogenous variables [40]. Structural equation modelling provides a very general framework for statistical analysis that includes several traditional multivariate procedures, sample factor analysis, regression analysis, differential analysis, and canonical correlation as special cases. SEM models are often visualised using a graphical path diagram. In contrast, a statistical model is usually represented in matrix equations [41].

It was possible to distinguish six variables in the model. Social ties diversity (STD) is the degree of diversity of the people that a user interacts with through social media [42]. Fake news awareness (FNA) is a social media user’s awareness of the existence of fake news on the online platforms they use [43]. Social media credibility (SMC) refers to the extent to which a reader believes that the information provided in social media is reliable, accurate, free from bias and complete [33]. Trust in people online (TPO) refers to the degree to which a person trusts other members of a network [44]. Information verification (IV) refers to the extent to which a user seeks to confirm the veracity of information seen on social media [35]. Intention to share (IS) information refers to the extent to which a user intends to share news [29].

A survey questionnaire was created using a Google form to collect data. The survey was published on the social networking site Facebook. Responses were collected in January 2021. Table 1 presents the collected sample of 245 responses, of which the majority were from women (58.4%). Most of the respondents were between the ages of 18 and 24 (64.9%), and the number of responses decreased with increasing age. Unfortunately, we only managed to collect one answer in the 45–54 age range and none from people over 55, which may be indicative of unfamiliarity with fake news or lack of interest in such topics. It is also worth noting the respondents’ education, which is directly related to age, as the highest percentage of respondents had secondary education (53.0%). Occupational status is also related to age and education, as the majority of respondents were pupils/students (64.1%). The high percentage of young people confirms the fact that this age group most frequently uses the internet and social media platforms and thus has the most contact with fake news. An additional segmentation question was also asked to ascertain on which social media platform the respondents most frequently come across fake news. The question was multiple choice. The vast majority of respondents confirmed the answer as Facebook (223 people), which is the social network with the highest number of active users. It is also worth mentioning Instagram, which was selected by 92 people. This may reflect the popular phenomenon in recent years of altering or retouching photos that are uploaded to this social platform.

## 4. Results

The SEM modelling was carried out using SmartPLS3 software [45]. As the structural model has many variables, the partial least squares of structural equation modelling (PLS-SEM) method was used to predict the key variables. In order to estimate the model, the following algorithms were employed: the PLS algorithm and Bootstrapping. The following settings were used to perform calculations for the PLS algorithm: a path-weighting scheme with the maximum number of iterations set to 1000; the stop criterion of 10^−X^ was set to 7. In the bootstrapping settings, 5000 subsamples were used in a two-tailed test type with bias correction and acceleration (BCa) at a significance level of 0.05. 

Table 2 presents the loadings for the individual variables. These have values above the threshold of 0.7, which indicates that they show an acceptable degree of reliability. The indicator reliability coefficients for all variables are also acceptable as they take values greater than 0.5.

The reliability of the measurement scales was assessed by calculating the Cronbach’s alpha score for each variable. The following items were removed to improve the model and results: FNA1, FNA4, SMC4, TPO4, and IS3. After recalculation, four variables had Cronbach’s alpha values above 0.70 and not exceeding 0.90, thus indicating adequate reliability (Table 3). In contrast, the remaining two variables are near the acceptable limit, so they can also be included in the model. The reliability, rho_A, as well as the composite reliability, also meet the required condition. In addition, convergence was assessed based on the average of variances extracted (AVE). All results exceeded the value of 0.50, which indicates that the individual elements explain most of the variance in their respective constructs and indicate acceptable convergent validity.

Discriminant validity means that two latent variables that represent different theoretical concepts are statistically different. The Heterotrait–monotrait ratio of correlations (HTMT) is a measure of similarity between latent variables. If the HTMT is clearly smaller than one, discriminant validity can be regarded as established. In many practical situations, a threshold of 0.85 reliably distinguishes between those pairs of latent variables that are discriminant valid and those that are not. Table 4 shows the discriminant validity assessment using the HTMT criterion [46]. All scores are below the acceptable threshold of 0.85. In view of the results, it can be concluded that the presence of discriminant validity has been established.

The estimated results show that the strongest relationships are between social ties diversity and fake news awareness and between fake news awareness and information verification. In Table 5, the T-statistics for all paths reach similar values and range from 2.35 to 4.084. The values of ƒ^2^ are relatively low because they do not exceed the threshold of 0.15. The path between fake news awareness and information verification shows the highest significance, which is 0.091. Therefore, the magnitude of the ƒ^2^ size effect shows that all paths have an impact but have low strength significance.

All *p*-values for the paths are less than 0.05 and can therefore be concluded to be significant. In summary, all the hypotheses were supported, indicating that the variables are influential.

Additional coefficients are included in Table 6 to show whether a variable is significant. Although the values of the R^2^ coefficient are relatively low, due to the relevant t-statistics, the R^2^ values can still be assumed significant. Furthermore, Q^2^ values are greater than zero for a particular variable and indicate acceptable predictive accuracy. Therefore, all the constructs included in Table 6 are significant as the Q^2^ range is from 0.024 to 0.249.

The model estimation results showed that the variables used in the model have a significant impact on the verification behaviour of internet users. This suggests that the verification of the SEM model estimated using SmartPLS3 was successful.

The results for the reflective model showed that the variables indicate a high level of reliability; the survey questions were constructed correctly without causing comprehension problems for the respondents. Table 3 shows the results for the reliability of the reflective variables, which also confirm adequate construct reliability. AVE testifies to the absence of measurement errors and explains the high degree of variance. An important criterion in the context of the whole model was to check the HTMT ratio in order to verify that the constructs do not link directly to each other. The values of this coefficient were found to be below the acceptable threshold of 0.85, indicating that there were no problems with discriminant validity. Therefore, the variables do not overlap and are fundamentally empirically different. 

Evaluation of the formative model’s results showed no problems with the indicators’ collinearity. After performing the Bootstrapping procedure in SmartPLS3, five items that showed low loading and were problematic for the model were removed. Thus, only those variables that had a significant impact on the research results were left in the final model.

Table 5 shows the results for each path. They are significant because the *p*-values for all paths are less than 0.05. In the structural analysis, the R^2^ and Q^2^ ratios were checked to confirm the results. Due to the respective t-statistics, the values of the R^2^ coefficient can be considered significant; all Q^2^ values are greater than zero, thus indicating acceptable predictive accuracy. 

The evaluation of the structural model confirmed the hypotheses that fake news awareness, social media credibility, trust in people online, and intention to share all influenced users’ information verification. Although all structural paths were significant, three showed a negative impact. This is because, in each case, a negative relationship between these variables was hypothesised. Therefore, trust in people negative impact on information verification is −0.238, social media credibility negative impact on information verification is −0.205, and fake news awareness negative impact on media credibility is −0.168. The results confirm the validity of the hypotheses between the variables and the directions of their relationships.

The two correlations had the greatest impact on each other. Fake news awareness affects information verification (0.267), and social ties diversity affects fake news awareness (0.270). Therefore, the main focus should be on flagging potential fake news and informing the public about the dangers of fake news. 

## 5. Discussion

In introducing the concept of fake news, a review of the available literature was carried out, and relevant aspects concerning this phenomenon were classified. The question regarding which social media platform users are most likely to encounter fake news was included in the survey. The significant prevalence of Facebook and Instagram shows that these two platforms should limit the spread of fake news [47].

As our study shows, we are faced with a flood of information. Fake news combating needs to be two-fold. On the one hand, there are several technical solutions to detect fake news on the web, such as machine learning and artificial intelligence [48]. On the other hand, internet users need to be more aware of fake news existing and possess some basic knowledge about fake news recognition [49]. Our results are in line with Trinicic et al. [50] and confirm that there is some lack of competence concerning media literacy [51]. Providing education in the field of digital media might offer a long-term solution for building resistance to fake news for future generations. 

The research makes important contributions to the further investigation of fake news. The results show that fake news verification behaviour is mainly driven by awareness of fake news. Therefore, it would be appropriate to focus on methods that alert users to the existence of fake news. A good solution would be to flag potential fake news in social media, which would generate more criticism among users who browse different sources. From a technical perspective, social media companies should design their websites to display alerts that inform users about potential fake news. If this were done, users would feel the need to verify information, or at least there would be a much smaller chance that they would believe such news. Consequently, the authors of fake news would become less popular, which would defeat their intended goal [39].

Some users are characterised by a lack of awareness of the dangers that careless use of social media can entail. From the very beginning, users are exposed to false or misleading news [16]. Social networking sites are very conducive to generating fake news. Facebook has estimated that up to 60 million bots have been created through their platform [2]. This is because some users browse social media without paying much attention to the content and fail to verify it in any way. 

Today, social media is considered by some people to be a reliable source of information, and it is increasingly common for many newspapers or trusted public institutions to provide information via their official social profiles on social media [52]. This is a very satisfactory solution for users, as it does not usually require payment and information is easily accessed. However, the development of social media has enabled the widespread distribution of fake news and a rise in the popularity of accounts that impersonate real profiles [14]. Research has shown that bots operating on social media platforms had a major impact on online discussions during the 2016 U.S. presidential election. In the week leading up to Election Day, an estimated 19 million fake accounts posted on Twitter about the presidential candidates D. Trump and H. Clinton. Their aim was to disrupt the online community during the election campaign and provoke users into emotional reaction and public discussion [14].

The appearance of fake news on Twitter is a particularly disturbing occurrence because it is a service valued by publicists and journalists. Such high-profile and trusted people use Twitter as a place to publish and as a source of knowledge and new information [53]. For every fact, there is a counter fact, and in the age of social media, the truth is becoming increasingly difficult to establish [54]. Furthermore, in some cases, counter-facts are compelling enough to effectively undermine the truth [55]. 

### 5.1. Theoretical Implications

There are several main reasons why social networks are actually a breeding ground for fake news. First, there are no security measures in place to control the quality of the content shared on such portals. Therefore, even without special software or tools, anyone can generate fake news. The second reason is the work of algorithms, which select the emerging content in such a way that it is directed towards the interests of a particular user. As a result, the information a user reads will generally be commensurate with their views, thus creating an information filter bubble [56]. Another factor is the popularity of social media and a large number of active daily users. The more people that use social media, the faster false information spreads. All this makes social media the main environment in which one comes across fake news. Therefore, it is all the more important to counteract this phenomenon. 

Fake news is a dangerous phenomenon, which is why there are so many different initiatives that aim to prevent the spread of fake news on the internet. These can be divided into two types of countermeasures: the first group aims to enable users to assess the veracity of information and recognise falsehoods; the second, by means of structural changes, aims to prevent and reduce the emergence of fake news [2].

An important aspect is the very awareness and understanding of the possibility of coming across fake news. There is a general need to educate users in this regard. Nowadays, young people have widespread and very easy access to news sources on the internet, but one must be careful about the quality of the sources from which information is drawn. This is a challenge for teachers and for the whole education system. In this regard, teaching should not only be limited to the ability to use specific internet tools, e.g., search engines: it must also focus on the issue of responsible use of the information that is available. Therefore, young people should be educated in verifying sources and selecting available information. The ability to critically analyse the content that is read is also important, as is understanding the principles of the algorithms that are responsible for the flow of information on the internet [23].

The ability to recognise fake news is important, but it is not easy. One must pay attention to many factors that may indicate that one is dealing with fake news. First, the user should look at the headline. If it is shocking and unlikely, there is a high chance that it is clickbait. The next step is to check the source, date, and author of this information. This makes it possible to verify whether the news comes from a trusted source or is of unknown origin. It is also advisable to check the history of the author’s account, e.g., when the site was created, how often the author publishes information, and what information has been published previously. Furthermore, attention should be paid to the photographs added to the published content, which can be manipulated or taken out of context. Authors of fake news often impersonate real websites by making small changes to the URL of the real website, so it is important to compare such questionable sources with the real ones [12].

When analysing the topic of fake news in social media, one should ask how online platforms help to reduce the spread of fake news. Social media could inform the audience about the quality of the source alongside a post that is being published. Platforms could also limit the spread of information by bots by means of excluding their activity from trend measurements. Therefore, a holistic data collection system would be needed to provide a dynamic understanding of the evolution of ubiquitous fake news delivery systems and thus mitigate them [2].

Currently, systems are being developed to help users detect and filter out potential fake news. They work by analysing a piece of information in terms of previously shared real and fake news [57]. More and more forms of checking the authenticity of information online are emerging. Examples include websites such as PolitiFact and Snopes, both of which analyse the veracity of news reports [2].

### 5.2. Practical Implications

From a practical point of view, news reporting companies and those running social media sites can use this study. In addition, the observations show how social ties affect the spread of fake news on social media. This research can provide preliminary information for both developers responsible for running social media sites and users who, in their own way, try to combat and limit the spread of fake news online [39].

The study group, which included young people, is also an important issue. The reason for the lack of response from older people may be due to a lack of interest or knowledge about fake news and thus an unwillingness to take part in the survey. The survey might also not have reached this demographic as it was shared on various groups in social networking sites, which are mostly used by young people. This is important information for those involved in informing the public about the existence of fake news. They should start publishing information in such a way that it also reaches older people, who may be completely unaware of and inadvertently influenced by this phenomenon.

### 5.3. Limitation and Future Research

The study does have some limitations. The investigated phenomenon of fake news did not consider a clear connection between the nature of the information and its recipient. Much of the fake news that appears on the internet is political in nature [58]. Therefore, a study that can check the relationship between fake news of a political character and its affiliation to a political group would be very valuable [59,60].

Data included in this study is subject to selection bias because people needed internet access to participate in the study and complete the survey, so the summarised evidence may not be fully generalisable to the entire population. This research does not exhaust the topic of the phenomenon of fake news; therefore, research should be conducted to show the influence of other factors on users’ verification of information on the internet, such as political affiliation, ideological views, or the perceived sense of security on social networking sites.

The sample of this study collected only from Polish social media users limits the generalisability of this study. Polish social media users’ perceptions of social media ties, credibility, and trust, may differ in different cultures. Therefore, future studies should consider cultural differences in examining social media users’ behavioural intentions. Finally, in this study, social media users’ fake news perceptions were measured instead of their actual behaviour. More specifically, this study was a perception-based study, and social media users’ actual fake news recognition was not examined. Therefore, future research studies utilising actual fact news recognition behaviour measures may offer more valid and accurate findings for social media and decision-makers in the internet publishing and marketing industry.

## 6. Conclusions

Fake news is a broad topic that is constantly evolving. This study aims to present and collect the assumptions that have been researched concerning this phenomenon and hopes to inspire further development of the issue of fake news. This is important because, to sum up the discussion so far, fake news has become a global problem. Fake news that is found on the internet influences personal and professional life and the political, cultural, and ideological spheres. The observations that have been made show the necessity of fighting fake news and finding better ways to limit its spread on the internet.

A major responsibility lies with social media creators, as most fake news appears on these sites. The larger the community, the more likely it is to encounter false information. Therefore, it is especially important for social media to introduce various solutions that would communicate the possibility of fake news. The need for this information is proved by the variables and their interrelationships, both of which have been studied in this work. As the strongest relationship was observed between fake news awareness and information verification, it is important that not only young internet users but also older people should be made aware of this threat. 

Fake news is a common and increasingly prevalent problem in online society. Through SEM modelling, it was possible to examine users’ information verification behaviour directly and check the strength and direction of the relationship between the remaining variables, which have a significant impact on the entire analysis. 

## Figures and Tables

**Table 1:** Summary of collected demographic responses from the survey.

Gender	Number of Respondents	Percentage
Female	143	58.4%
Male	102	41.6%
**Age**	**Number of respondents**	**Percentage**
Less than 18 years	7	2.9%
18–24 years	159	64.9%
25–34 years	60	24.5%
35–44 years	18	7.3%
45–54 years	1	0.4%
55–64 years	0	0%
65 years and over	0	0%
**Education**	**Number of respondents**	**Percentage**
Primary education	11	4.5%
Vocational education	6	2.4%
Secondary Education	132	53.9%
Higher education	96	39.2%
**Professional status**	**Number of respondents**	**Percentage**
Pupil/student	157	64.1%
Employed full-time	63	25.7%
Part-time employee	15	6.1%
Not employed	10	4.1%
**The social platform where you most often come across fake news**	**Number of respondents**	**Percentage**
Facebook	223	91%
Instagram	92	37.6%
Snapchat	15	6.1%
Twitter	39	15.9%
Wykop.pl	29	11.8%
Reddit	10	4.1%

**Table 2:** The relevance of reflective variables.

Variable	Item	Loadings	Indicator Reliability	AVE
		>0.7	>0.5	>0.5
FNA	FNA2	0.891	0.794	0.584
	FNA3	0.856	0.750	
	FNA5	0.864	0.747	
IS	IS1	0.959	0.920	0.887
	IS2	0.981	0.963	
SMC	SMC1	0.943	0.891	0.812
	SMC2	0.948	0.900	
	SMC3	0.954	0.912	
STD	STD1	0.923	0.853	0.585
	STD2	0.871	0.759	
	STD3	0.819	0.672	
TPO	TPO1	0.924	0.855	0.713
	TPO2	0.918	0.843	
	TPO3	0.913	0.834	
IV	IV1	0.902	0.814	0.673
	IV2	0.846	0.717	
	IV3	0.916	0.840	
	IV4	0.948	0.900	
	IV5	0.904	0.818	

**Table 3:** Reliability of reflective variables.

Construct	Cronbach’s Alpha	Reliability ρA (rho_A)	Composite Reliability
	0.7–0.9	>0.7	>0.7
FNA	0.701	0.754	0.808
IS	0.877	0.963	0.940
SMC	0.884	0.887	0.928
STD	0.703	0.755	0.808
TPO	0.806	0.840	0.881
IV	0.877	0.891	0.911

**Table 4:** HTMT values.

	FNA	IS	SMC	STD	TPO
**IS**	0.049				
**SMC**	0.232	0.219			
**STD**	0.372	0.085	0.182		
**TPO**	0.274	0.225	0.470	0.176	
**IV**	0.451	0.155	0.347	0.407	0.393

**Table 5:** Path coefficients.

Hypothesis	Path	Path Coefficient	T-Statistics	ƒ^2^	*p*-Value < 0.05	Hypothesis Supported
**H1**	STD → FNA	0.270	3.715	0.079	0.000	Yes
**H2**	TPO → IV	−0.238	3.561	0.061	0.000	Yes
**H3**	FNA → SMC	−0.168	2.356	0.029	0.018	Yes
**H4**	FNA → IV	0.267	4.084	0.091	0.000	Yes
**H5**	SMC → IV	−0.205	2.859	0.046	0.004	Yes
**H6**	IS → IV	0.191	2.534	0.046	0.011	Yes

**Table 6:** The size of R^2^ and Q^2^ indicators.

Variable	R^2^	Q^2^
**IV**	0.262	0.249
**SMC**	0.028	0.024
**FNA**	0.073	0.069

## Data Availability

The data presented in this study are available on request from the corresponding author. The data are not publicly available due to privacy.

## Appendix A

**Table A1:** Survey questions.

Variable	Item	Question
Social ties diversity	STD1	The people I interact with through social media represent the different groups I am involved in.
	STD2	The people I interact with through social media represent many stages of my life.
	STD3	The people I interact with through social media are diverse in terms of how I met them.
Fake news awareness	FNA1	I am aware of the existence of fake news and the social consequences it entails.
	FNA2	I am concerned about the phenomenon of fake news.
	FNA3	I am aware that I may come across fake news when using social media.
	FNA4	I have sufficient knowledge about fake news and its social impact.
	FNA5	I understand the concerns about fake news and its negative impact on society.
Social media credibility	SMC1	I believe that most of the news that is published on social networks is credible.
	SMC2	I believe that most of the news that is published on social networks is relevant/accurate.
	SMC3	I believe that most of the news that is published on social networks is trustworthy.
	SMC4	I believe that most of the news that is published on social networks contains all the information on a topic.
Trust in people online	TPO1	It is easy for me to trust another person on the internet.
	TPO2	My tendency to trust another person online is high.
	TPO3	I tend to trust people who publish information on the internet even though I have little knowledge of the subject.
	TPO4	Trusting someone or something on the internet is not difficult.
Information verification	IV1	I check who the author is of the news I see on social media.
	IV2	I look for official confirmation of information or a recommendation from someone I know to verify news that is published on social media.
	IV3	I pay attention to whether published information on social media has a stated source.
	IV4	I verify the author of published information or news I see.
	IV5	I consider the purpose of the information published by an author.
Intention to share	IS1	In the future, I intend to share news on social networks.
	IS2	I intend to share news regularly on social networks.
	IS3	I expect to share news with other users on social media.

## References

[1] Gelfert A. (2018). Fake News: A Definition. Informal Log..

[2] Lazer D.M.J., Baum M.A., Benkler Y., Berinsky A.J., Greenhill K.M., Menczer F., Metzger M.J., Nyhan B., Pennycook G., Rothschild D. (2018). The science of fake news. Science.

[3] Flintham M., Karner C., Bachour K., Creswick H., Gupta N., Moran S. (2018). Falling for Fake News. Proceedings of the 2018 CHI Conference on Human Factors in Computing Systems.

[4] Wardle C., Derakhshan H. (2018). Thinking about ‘information disorder’: Formats of misinformation, disinformation, and mal-information. Handbook for Journalism Education and Training UNESCO Series on Journalism Education.

[5] Naeem S.B., Bhatti R., Khan A. (2020). An exploration of how fake news is taking over social media and putting public health at risk. Heal. Inf. Libr. J..

[6] Vosoughi S., Roy D., Aral S. (2018). The spread of true and false news online. Science.

[7] Ong J.C., Cabañes J.A.V. (2018). Architects of Networked Disinformation: Behind the Scenes of Troll Accounts and Fake News Production in the Philippines. Archit. Netw. Disinformation Scenes Troll Acc. Fake News Prod. Philipp..

[8] Allcott H., Gentzkow M. (2017). Social Media and Fake News in the 2016 Election. J. Econ. Perspect..

[9] Spohr D. (2017). Fake news and ideological polarization. Bus. Inf. Rev..

[10] Grinberg N., Joseph K., Friedland L., Swire-Thompson B., Lazer D. (2019). Fake news on Twitter during the 2016 U.S. presidential election. Science.

[11] McNair B. (2017). Fake News.

[12] Molina M.D., Sundar S.S., Le T., Lee D. (2021). “Fake News” Is Not Simply False Information: A Concept Explication and Taxonomy of Online Content. Am. Behav. Sci..

[13] Gorwa R., Guilbeault D. (2020). Unpacking the Social Media Bot: A Typology to Guide Research and Policy. Policy Internet.

[14] Shu K., Sliva A., Wang S., Tang J., Liu H. (2017). Fake News Detection on Social Media. ACM SIGKDD Explor. Newsl..

[15] Gorwa R. (2017). Computational Propaganda in Poland: False Amplifiers and the Digital Public Sphere.

[16] Shao C., Ciampaglia G.L., Varol O., Yang K.-C., Flammini A., Menczer F. (2018). The spread of low-credibility content by social bots. Nat. Commun..

[17] Kalveks T. (2018). Clickbait. The Blackwell Encyclopedia of Sociology.

[18] Khater S.R., Al-sahlee O.H., Daoud D.M., El-Seoud M.S.A. (2018). Clickbait Detection. Proceedings of the 7th International Conference on Software and Information Engineering—ICSIE ’18.

[19] Chakraborty A., Paranjape B., Kakarla S., Ganguly N. (2016). Stop Clickbait: Detecting and preventing clickbaits in online news media. Proceedings of the 2016 IEEE/ACM International Conference on Advances in Social Networks Analysis and Mining (ASONAM).

[20] Zheng H.-T., Chen J.-Y., Yao X., Sangaiah A., Jiang Y., Zhao C.-Z. (2018). Clickbait Convolutional Neural Network. Symmetry.

[21] Probierz B., Stefański P., Kozak J. (2021). Rapid detection of fake news based on machine learning methods. Procedia Comput. Sci..

[22] Probierz B., Kozak J., Stefański P., Juszczuk P. (2021). Adaptive Goal Function of Ant Colony Optimization in Fake News Detection.

[23] Flaxman S., Goel S., Rao J.M. (2016). Filter Bubbles, Echo Chambers, and Online News Consumption. Public Opin. Q..

[24] DiFranzo D., Gloria-Garcia K. (2017). Filter bubbles and fake news. XRDS Crossroads ACM Mag. Stud..

[25] Buckels E.E., Trapnell P.D., Paulhus D.L. (2014). Trolls just want to have fun. Personal. Individ. Differ..

[26] Daskal E., Wentrup R., Shefet D. (2020). Taming the Internet Trolls With an Internet Ombudsperson: Ethical Social Media Regulation. Policy Internet.

[27] Zubiaga A., Ji H. (2014). Tweet, but verify: Epistemic study of information verification on Twitter. Soc. Netw. Anal. Min..

[28] Grabner-Kräuter S., Bitter S. (2015). Trust in online social networks: A multifaceted perspective. Forum Soc. Econ..

[29] Lee C.S., Ma L. (2012). News sharing in social media: The effect of gratifications and prior experience. Comput. Hum. Behav..

[30] Gawron M., Strzelecki A. (2021). Consumers’ Adoption and Use of E-Currencies in Virtual Markets in the Context of an Online Game. J. Theor. Appl. Electron. Commer. Res..

[31] Rieh S.Y. (2002). Judgment of information quality and cognitive authority in the Web. J. Am. Soc. Inf. Sci. Technol..

[32] Scheufele D.A., Krause N.M. (2019). Science audiences, misinformation, and fake news. Proc. Natl. Acad. Sci. USA.

[33] Flanagin A.J., Metzger M.J. (2007). The role of site features, user attributes, and information verification behaviors on the perceived credibility of web-based information. New Media Soc..

[34] Cooke N.A. (2017). Posttruth, Truthiness, and Alternative Facts: Information Behavior and Critical Information Consumption for a New Age. Libr. Q..

[35] Flanagin A.J., Metzger M.J. (2000). Perceptions of Internet Information Credibility. J. Mass Commun. Q..

[36] Van Duyn E., Collier J. (2019). Priming and Fake News: The Effects of Elite Discourse on Evaluations of News Media. Mass Commun. Soc..

[37] Lukowicz K., Strzelecki A. (2020). User Satisfaction on Social Media Profile of E-sports Organization. Mark. Manag. Innov..

[38] Vitak J., Kim J. You can’t block people offline. Proceedings of the 17th ACM Conference on Computer Supported Cooperative Work & Social Computing.

[39] Torres R., Gerhart N., Negahban A. (2018). Epistemology in the Era of Fake News: An Exploration of Information Verification Behaviors among Social Networking Site Users. ACM SIGMIS Database Database Adv. Inf. Syst..

[40] Vinodh S., Joy D. (2012). Structural Equation Modelling of lean manufacturing practices. Int. J. Prod. Res..

[41] Hox J.J., Bechger T.M. (1999). An introduction to structural equation modeling. Stud. Health Technol. Inform..

[42] Gerhart N., Sidorova A. (2017). The Effect of Network Characteristics on Online Identity Management Practices. J. Comput. Inf. Syst..

[43] Bulgurcu C. (2010). Benbasat Information Security Policy Compliance: An Empirical Study of Rationality-Based Beliefs and Information Security Awareness. MIS Q..

[44] McKnight D.H., Chervany N.L. (2001). Conceptualizing trust: A typology and e-commerce customer relationships model. Proceedings of the 34th Annual Hawaii International Conference on System Sciences.

[45] Ringle C.M., Wende S., Becker J.-M. (2015). SmartPLS 3.

[46] Henseler J., Ringle C.M., Sarstedt M. (2015). A new criterion for assessing discriminant validity in variance-based structural equation modeling. J. Acad. Mark. Sci..

[47] Lenartowicz M., Strzelecki A. (2021). Moderate Effect of Satisfaction on Intention to Follow Business Profiles on Instagram. Int. J. Mark. Commun. New Media.

[48] Juszczuk P., Kozak J., Dziczkowski G., Głowania S., Jach T., Probierz B. (2021). Real-World Data Difficulty Estimation with the Use of Entropy. Entropy.

[49] Pilgrim J., Vasinda S. (2021). Fake News and the “Wild Wide Web”: A Study of Elementary Students’ Reliability Reasoning. Societies.

[50] Trninić D., Kuprešanin Vukelić A., Bokan J. (2021). Perception of “Fake News” and Potentially Manipulative Content in Digital Media—A Generational Approach. Societies.

[51] Cicha K., Rutecka P., Rizun M., Strzelecki A. (2021). Digital and Media Literacies in the Polish Education System—Pre- and Post-COVID-19 Perspective. Educ. Sci..

[52] Iosifidis P. (2011). The public sphere, social networks and public service media. Inf. Commun. Soc..

[53] Bail C.A., Guay B., Maloney E., Combs A., Hillygus D.S., Merhout F., Freelon D., Volfovsky A. (2020). Assessing the Russian Internet Research Agency’s impact on the political attitudes and behaviors of American Twitter users in late 2017. Proc. Natl. Acad. Sci. USA.

[54] Gary R. Lies, Propaganda and Fake News: A Challenge for our Age. BBC News.

[55] Fitzpatrick N. (2018). Media Manipulation 2.0: The Impact of Social Media on News, Competition, and Accuracy. Athens J. Mass Media Commun..

[56] Seargeant P., Tagg C. (2019). Social media and the future of open debate: A user-oriented approach to Facebook’s filter bubble conundrum. Discourse Context Media.

[57] Rubin V.L., Chen Y., Conroy N.K. (2015). Deception detection for news: Three types of fakes. Proc. Assoc. Inf. Sci. Technol..

[58] Calvillo D.P., Garcia R.J.B., Bertrand K., Mayers T.A. (2021). Personality factors and self-reported political news consumption predict susceptibility to political fake news. Pers. Individ. Dif..

[59] Calvillo D.P., Rutchick A.M., Garcia R.J.B. (2021). Individual Differences in Belief in Fake News about Election Fraud after the 2020 U.S. Election. Behav. Sci..

[60] Calvillo D.P., Ross B.J., Garcia R.J.B., Smelter T.J., Rutchick A.M. (2020). Political Ideology Predicts Perceptions of the Threat of COVID-19 (and Susceptibility to Fake News About It). Soc. Psychol. Personal. Sci..

